# Functional Role of G9a Histone Methyltransferase in Cancer

**DOI:** 10.3389/fimmu.2015.00487

**Published:** 2015-09-25

**Authors:** Francesco Casciello, Karolina Windloch, Frank Gannon, Jason S. Lee

**Affiliations:** ^1^Control of Gene Expression Laboratory, QIMR Berghofer Medical Research Institute, Herston, QLD, Australia; ^2^School of Natural Sciences, Griffith University, Nathan, QLD, Australia; ^3^Faculty of Health, School of Biomedical Sciences, Queensland University of Technology, Kelvin Grove, QLD, Australia; ^4^School of Chemistry and Molecular Biosciences, University of Queensland, Brisbane, QLD, Australia

**Keywords:** histone methylation, epigenetic regulation, cancer, G9a, tumor growth, metastasis

## Abstract

Post-translational modifications of DNA and histones are epigenetic mechanisms, which affect the chromatin structure, ultimately leading to gene expression changes. A number of different epigenetic enzymes are actively involved in the addition or the removal of various covalent modifications, which include acetylation, methylation, phosphorylation, ubiquitination, and sumoylation. Deregulation of these processes is a hallmark of cancer. For instance, G9a, a histone methyltransferase responsible for histone H3 lysine 9 (H3K9) mono- and dimethylation, has been observed to be upregulated in different types of cancer and its overexpression has been associated with poor prognosis. Key roles played by these enzymes in various diseases have led to the hypothesis that these molecules represent valuable targets for future therapies. Several small molecule inhibitors have been developed to specifically block the epigenetic activity of these enzymes, representing promising therapeutic tools in the treatment of human malignancies, such as cancer. In this review, the role of one of these epigenetic enzymes, G9a, is discussed, focusing on its functional role in regulating gene expression as well as its implications in cancer initiation and progression. We also discuss important findings from recent studies using epigenetic inhibitors in cell systems *in vitro* as well as experimental tumor growth and metastasis assays *in vivo*.

## Introduction

Cancer is a heterogeneous disease, commonly believed to solely arise from the acquisition of genetic mutations, leading to a loss of functionality of genes that prevent uncontrolled cell growth (tumor suppressor genes), as well as to a deregulated activity of genes that promote proliferation (oncogenes). However, for a cell, it is not only important to possess functional genes, but it is also fundamental to express them appropriately to maintain a normal phenotype. Processes that regulate gene expression include epigenetic mechanisms such as DNA and histone modifications, which have been found to be often deregulated in different types of cancer. Different epigenetic modifying enzymes can actively promote or remove modifications directly on the DNA or on specific residues on histone tails, regulating various chromatin-related processes (1–3).

The term “epigenetic” was initially introduced by Conrad Waddington in 1940, defining it as the branch of biology studying the interactions between genes and their products (4). In time, the definition has narrowed and it is nowadays used to identify heritable and long-term changes in gene expression that do not necessarily involve mutations in DNA sequences (1).

**Figure 1:**
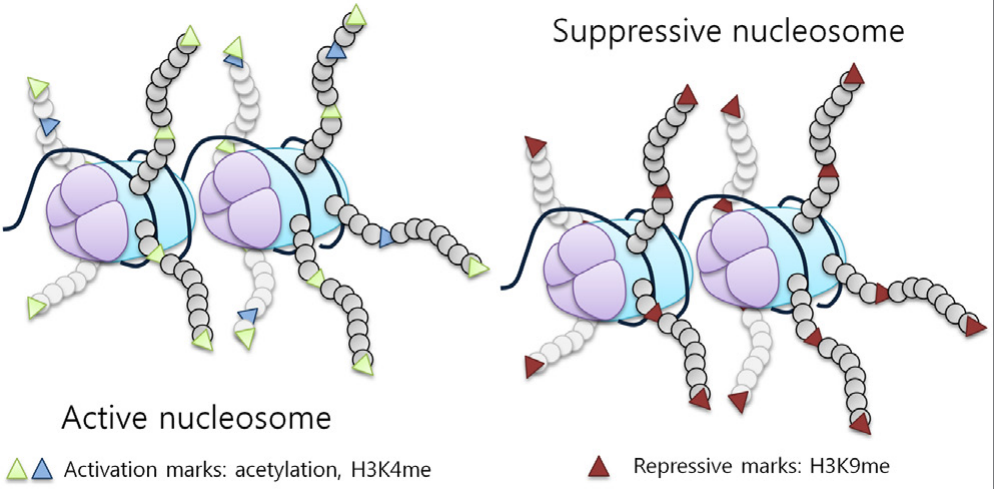
**Histone modifications influence chromatin structure and activity**. Histone tail modifications lead to a change in histone affinity for the DNA, causing the chromatin to shift between an open (active) and a closed (suppressive) state. An example of a repressive mark is H3K9 methylation, while activation marks correspond to acetylation and H3K4 methylation [adapted from Biran et al. (7)].

DNA is packaged into chromatin by histones forming nucleosomes. The nucleosome is organized around an octamer composed of two molecules of each histone protein, H2A, H2B, H3, and H4, with 145 base pairs of DNA wrapped around it (5). Histones are essential proteins characterized by a globular carboxy-terminal domain and a protruding, lysine rich, N-terminal tail. The N-terminal tails of histones are subject to reversible covalent modifications, which ultimately affect gene expression. Histones can be modified by an array of post-translational modifications including acetylation, methylation, phosphorylation, ubiquitination, and sumoylation (6). These modifications regulate the ability of transcription factors to access the underlying DNA by modifying histone affinity for its negatively charged sugar backbone, representing a fundamental regulatory mechanism, which is able to impact transcription (Figure 1), replication, and chromatin stability (7, 8).

## Histone Modifications

### Acetylation

Acetylation refers to the addition of an acetyl group at lysine residues in the N-terminal tails of histones. The effect is to neutralize the positive charge of the histone tails, hence promoting the opening of DNA and increasing its accessibility to transcription factors (Figure 1, active nucleosome) (9, 10). Histone acetylation is regulated by two classes of enzymes, histone acetyltransferases (HATs) and deacetylases (HDACs). Different proteins, which display this intrinsic activity are components of the RNA polymerase II complex or proteins that associate with transcription factors (11).

### Phosphorylation

Like other proteins, histones are phosphorylated by kinases and are involved in the DNA damage repair mechanism, chromatin compaction, and transcriptional regulation (12). DNA damage repair has been associated with phosphorylation at serine 139 (S139) on H2. In yeast, it has been shown that this residue is phosphorylated following DNA damage. This modification spreads for several kilobases on each side of the break on the DNA and it is thought to recruit DNA damage repair factors, such as the mediator of DNA damage checkpoint protein 1 (MDC1), promoting the recruitment of DNA repair proteins at the site of the damage (13–15). On the other hand, phosphorylation of H3 in mammals was shown to be necessary for chromatin compaction, promoting proper chromosomal condensation, and segregation. Moreover, studies have demonstrated that H3 phosphorylation is also able to affect gene expression, inducing transcription, through a crosstalk between other types of histone modifications (acetylation and methylation) (16, 17). Recently, many kinases thought to have only cytoplasmic function were shown to phosphorylate histones in the nucleus to affect gene expression (18, 19).

### Ubiquitination and sumoylation

Histones undergo ubiquitination, however, unlike many other proteins; ubiquitination of histones does not lead to proteasomal degradation. Ubiquitin rather acts as a signaling molecule and either its addition or removal from histones can be associated with transcriptional activation. Cross-talk between ubiquitination and methylation has been observed, suggesting that ubiquitination and deubiquitination-related effects are mainly mediated by influencing the histone methylation status (20). Small ubiquitin-related modifier (SUMO) is a family of ubiquitin-like proteins, generally involved in post-translational modifications. SUMO proteins resemble ubiquitin both in their structure and in their ligation mechanism, but their addition to histones leads to different consequences in respect to ubiquitination. In fact, sumoylation has been associated with transcriptional repression (6, 21). It has also been shown recently that sumoylation decreases the affinity between two adjacent nucleosomes, suggesting that this modification might influence gene expression without involving chromatin compaction (22).

### Histone methylation

Histone methylation, similar to DNA methylation, has been generally associated with gene repression (23). However, it is known that several lysine methylation patterns can also characterize active genes, such as tri-methylated H3K4 or H3K9 mono-methylation (24, 25). Histones are methylated by several histone methyltransferases (HMTs) and methylation is actively removed by histone demethylases (HDMs). The functional role of histone methylation and its implications in cancer will be discussed in detail in the next section.

Methylation of histone tails occurs at either lysine or arginine residues, on histones H3 and H4, and, although normally associated with gene silencing, specific methylation sites are known to correlate with active promoters (26–28). For instance, methylation of histones H3K4, H3K36, and H3K79 is associated with gene activation (28, 29), while methylation on H3K9 or H3K27 residues associates with transcriptional repression. On histone H4, K20 methylation is a known mark of gene silencing (27, 28). Similarly to lysine methylation, arginine methylation has been linked to both gene activation (H3R17) and repression (H3R2, H4R3) (30–32).

Lysines can be mono-, di- and trimethylated, whereas arginines can only be mono- or dimethylated. The fact that gene expression was regulated by methylation has been known previously; the discovery of HMT, SUV39H1, has facilitated the understanding of histone methylation and gene expression (33, 34). SUV39H1, also known as KMT1A, is a lysine methyltransferase, conserved from yeast to human, and is a homolog of the *Drosophila* methyltransferase Su(var) 3-9 (33). KMT1A is characterized by the presence of a SET domain, which is a 130 amino acid long catalytic domain, initially found to be conserved in Su(var) 3-9, Enhancer of zeste (Ez) and trithorax (27). Other lysine methyltransferases have been identified by homology to this domain, and altogether form the larger family of lysine methyltransferases (KMTs). Protein arginine methyltransferases (PRMTs), on the other hand, catalyze the transfer of methyl groups on arginine residues. Several arginine methyltransferases have been shown to methylate histone and non-histones to affect gene expression in various contexts (28, 35).

Methylation at different histone residues are associated with either repressive or active chromatin states (36). For instance, while H3K9 di- and trimethylation are transcriptional repressive marks, H3K9 mono-methylation has been observed to characterize active promoters (25). It appears that the cells are able to respond to different histone modifications through various chromatin-associated proteins, which target specific modifications on histone residues, such as the repressive heterochromatin protein 1 (HP1), leading to different expression patterns. HP1 binds to methyl groups on histone H3K9 for gene repression (37). On the other hand, other factors, such as the transcriptional activator WDR5 promote gene activation. WDR5 recognizes methylated H3K4, a modification associated with active promoters (38).

Antagonists of HMTs are enzymes, which remove the methyl mark from histones, known as HDMs. The first identified was peptidylarginine deiminase 4 (PADI4), which reversed arginine methylation (39). Lysine demethylation is instead carried out by lysine-specific demethylase 1 (LSD1) and the next large class of enzymes identified was the Jumonji C (JmjC) domain containing demethylases (15, 40). While LSD1 can only remove mono- and dimethyl modifications, JmjC domain-containing enzymes were shown to remove all three methylation marks (15).

Histone methylation plays key roles in different processes other than gene expression regulation, such as imprinting and chromosome stability (41). Being an important regulatory mechanism of gene expression, it is not surprising that its deregulation has been implicated in various types of cancer, such as breast, prostate, lung, and brain. Moreover, patterns of histone methylation have been found to be severely altered in cancer cells, and this can involve both a gain and a loss of histone methylation (42).

### Crosstalk in histone modifications

As discussed earlier, histones can be modified by various processes, ultimately leading to different patterns of gene expression. An addition of complexity to this already complex system is brought about by the fact that certain residues may accept multiple modifications. For instance, lysine residue (K) can be targeted for distinct modifications such as acetylation, methylation, ubiquitination, or sumoylation and can harbor one, two, or three methyl residues. Moreover, specific histone modifications were shown to promote the generation or the loss of other modifications, demonstrating the existence of a crosstalk between them. Following this evidence, it is thought that the particular combination of N-terminal modifications results in specific signals which the cell is able to interpret as a readable code, known as the “histone code” (43, 44).

**Figure 2:**
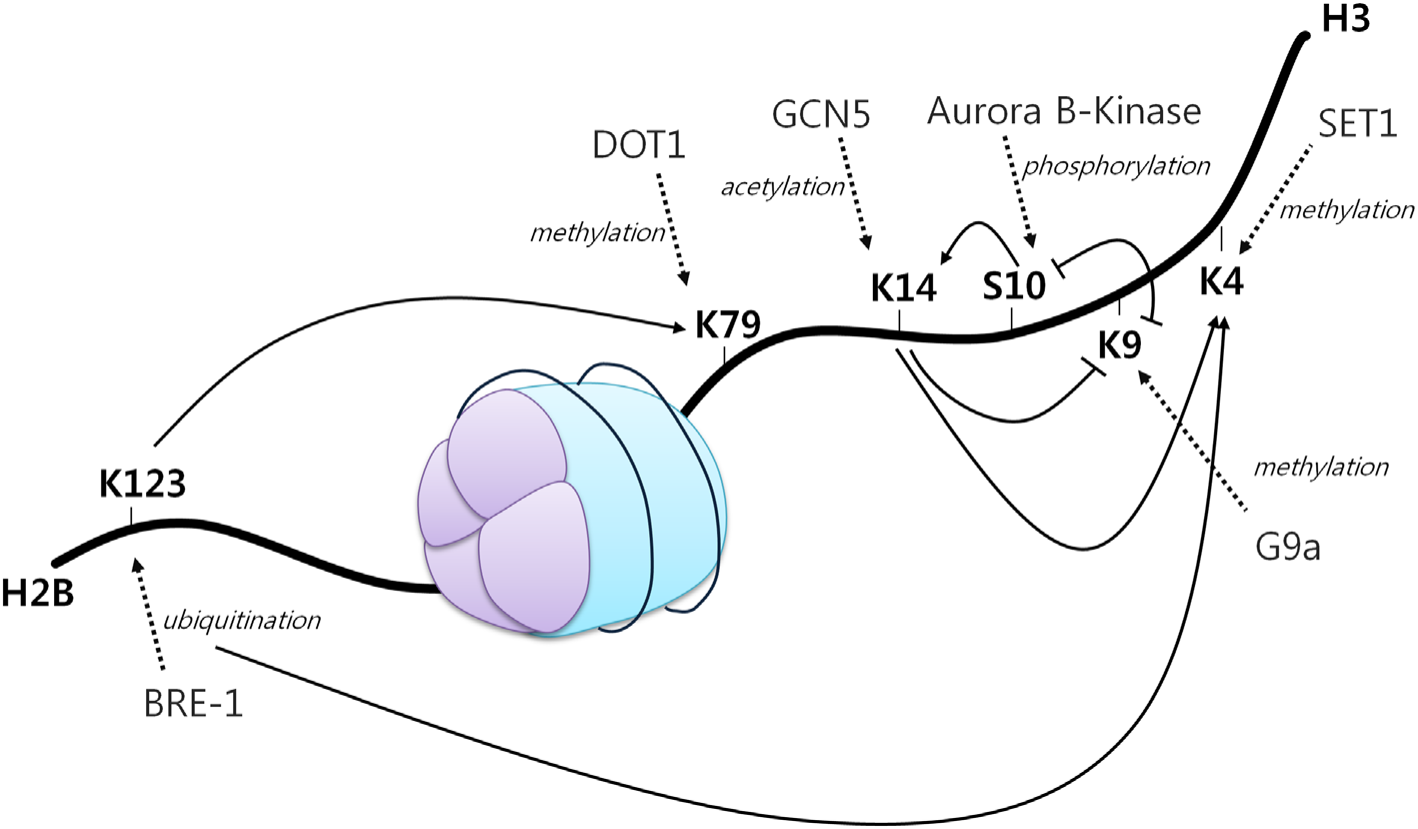
**The histone code**. A core histone showing modifications on two different histone tails (H2B and H3). Different histone modifications can positively or negatively influence the generation of others, mediating a complex crosstalk influencing gene expression. Arrowheads indicate positive effects while flat heads indicate negative effects. Dotted arrows display different enzymes, their function and site of action [adapted from Bannister et al. (43)].

A first example of histone crosstalk is shown by the relationship between H3S10 phosphorylation and H3K14 acetylation (Figure 2). It has been observed that phosphorylation of this serine residue induces the HAT Gcn5 to acetylate K14 on H3. H3S10 phosphorylation was also demonstrated to inhibit H3K9 modifications (45). Other studies have also demonstrated the requirement of H2BK123 monoubiquitination for H3K4 and H3K79 methylation. Specifically, H2BK123 ubiquitination is dispensable for monomethylation of the other two residues, but it is necessary for their di- and trimethylation (46). In addition, it has been shown that a point mutation in H3K14 results in a specific loss of H3K4 trimethylation, but not mono and dimethylation. As H3K14 is a known acetylation site, thus revealing its requirement for H3K4 trimethylation (47).

A number of different types of crosstalk are present in the cellular context, involving different combinations of histone modifications, and these can occur at various regions across the genome. Understanding the complex language of histones is the key in comprehending the events which regulate gene expression.

## Functional Role of G9a in Regulating Gene Expression

### G9a as a histone methyltransferase

**Figure 3:**
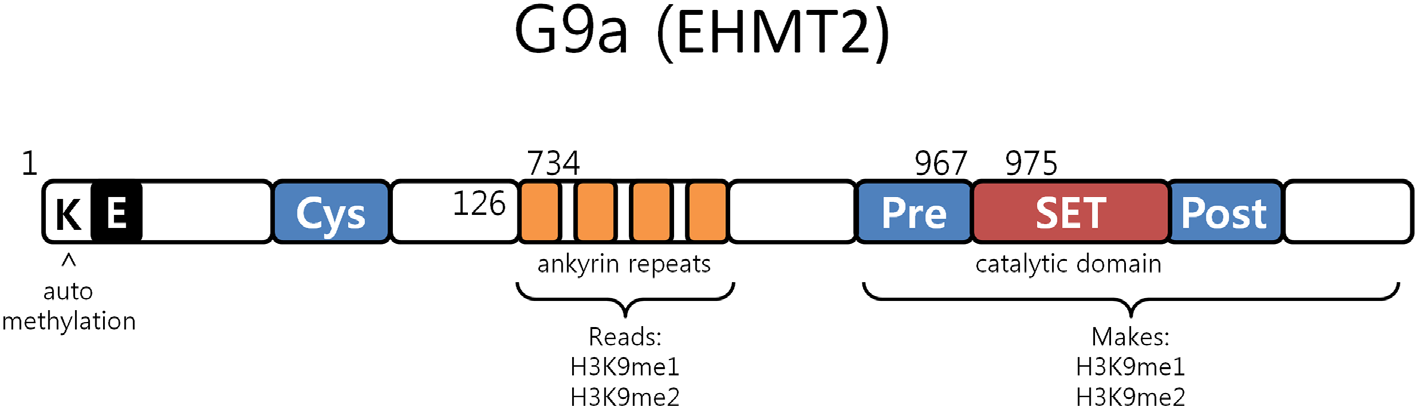
**G9a structure**. G9a structural organization characterized by an automethylation site at its N-terminal end, ankyrin repeats which recognize mono and dimethylated histone H3K9 and by a catalytic SET domain, responsible for the enzymatic activity [adapted from Collins et al. (50)].

G9a is a nuclear histone lysine methyltransferase (HMT) belonging to the Su(var)3-9 family, which mainly catalyzes histone H3 lysine 9 mono- and dimethylation, a reversible modification generally associated with transcriptional gene silencing. Structurally, it is composed of a catalytic SET domain, a domain containing ankyrin repeats (involved in protein–protein interactions) and nuclear localization signals on the N-terminal region (Figure 3) (47–50). G9a SET domain is responsible for the addition of methyl groups on H3, whereas the ankyrin repeats have been observed to represent mono- and dimethyl lysine binding regions. G9a is thus not only able to both methylate histone tails but also able to recognize this modification, functioning as a scaffold for the recruitment of other target molecules on the chromatin (46).

A G9a-like protein (GLP) has also been identified, which actively interacts with G9a, forming a heterodimeric complex. It has been shown that the heteromeric structure is the predominant form, as well as the active state, of the methyltransferase *in vivo* (51). However, recent studies have also demonstrated that while the heterodimer seems to be required for G9a–GLP methyltransferase activity, the enzymatic activity of G9a is more important for the *in vivo* function of the complex (23). The complex is responsible for methylating H3K9, a mark which is recognized by the heterochromatin protein 1 (HP1), leading to transcriptional silencing (52).

The genetic manipulation of G9a in mice has yielded G9a-deficient embryonic stem (ES) cells allowing studies determining the functional role of G9a in development. G9a-deficient cells were characterized by a loss of global methylation of chromatin, but not at heterochromatic regions. While HMT activity can generally be correlated with both heterochromatin organization and euchromatin, this result demonstrated that G9a is a unique enzyme that can specifically associate with euchromatin and, thus, involved in the repression of active promoters (51, 53, 54). Its repressive activity was revealed to be fundamental in embryogenesis in mice. G9a depletion resulted in embryonic lethality with severe differentiation defects in ES cells, demonstrating that G9a is essential for the repression of developmental genes and that it is required during development (55). Moreover, G9a was also found to be involved in the acquisition of cell specification, as a necessary factor for the inhibition of the *Oct-3/4*, a homeobox gene important for the maintenance of pluripotency (56). In the silencing of *Oct-3/4*, G9a histone methylation activity synergizes with DNA methylation, to induce a long term repression of the gene. Following G9a-mediated histone methylation, HP1 is recruited to the methylated site, preventing transcription. A DNA methyltransferase, DNMT1, is then recruited at the site, promoting methylation of nearby sites on the DNA, reinforcing the inhibitory signal (56). This demonstrates that histone methylation and DNA methylation, although carried out by different enzymes, possess a close biological relationship, cooperatively mediating gene repression through a system which could be defined as a “double lock” (1). Moreover, G9a was also reported to represent a negative regulator of pathogenic T-cell differentiation as G9a depleted T-cells were shown to possess an increased sensitivity to TGF-β1, promoting naive T-cell differentiation in pathogenic T-cells, Th17 and Treg, in the absence of intestinal inflammation (57). This essentially demonstrated that G9a expression is a key factor in the maintenance of T-cell homeostasis. G9a has also been shown to be involved in the T-cell differentiation process. CD4^+^ T-cells, for instance, fail to differentiate into Th2 cells both *in vitro* and *in vivo* in the absence of G9a. Mice carrying a T-cell-specific G9a deletion could not develop Th2 cells in response to infection in the absence of interferon-γ (IFN-γ). In addition, CD4^+^ T-cells from wild type mice, when stimulated under normal, Th1 and Th2 conditions, were characterized by an increased expression of IL-17A after pharmacologic inhibition of G9a (58). Precursor lymphocytes are known to undergo a unique re-arrangement of the genes that encode different antigen receptors of B and T lymphocytes, through a process called V(D)J recombination, mediating the assembly of immunoglobulin (Ig) and T-cell receptor (TCR). In this context, G9a recruitment inhibits transcription and recombination of adjacent gene segments, inducing gene silencing and promoting DNA hypermethylation (59). However, G9a inhibition has been shown to not affect lymphocyte development and V(D)J recombination, only displaying a slight impairment in the usage of Igγ L chains (60). This could be explained by the fact that DNA methylation and histone methylation cooperate, and the silencing of only G9a does not completely block DNA methylation and gene repression.

**Figure 4:**
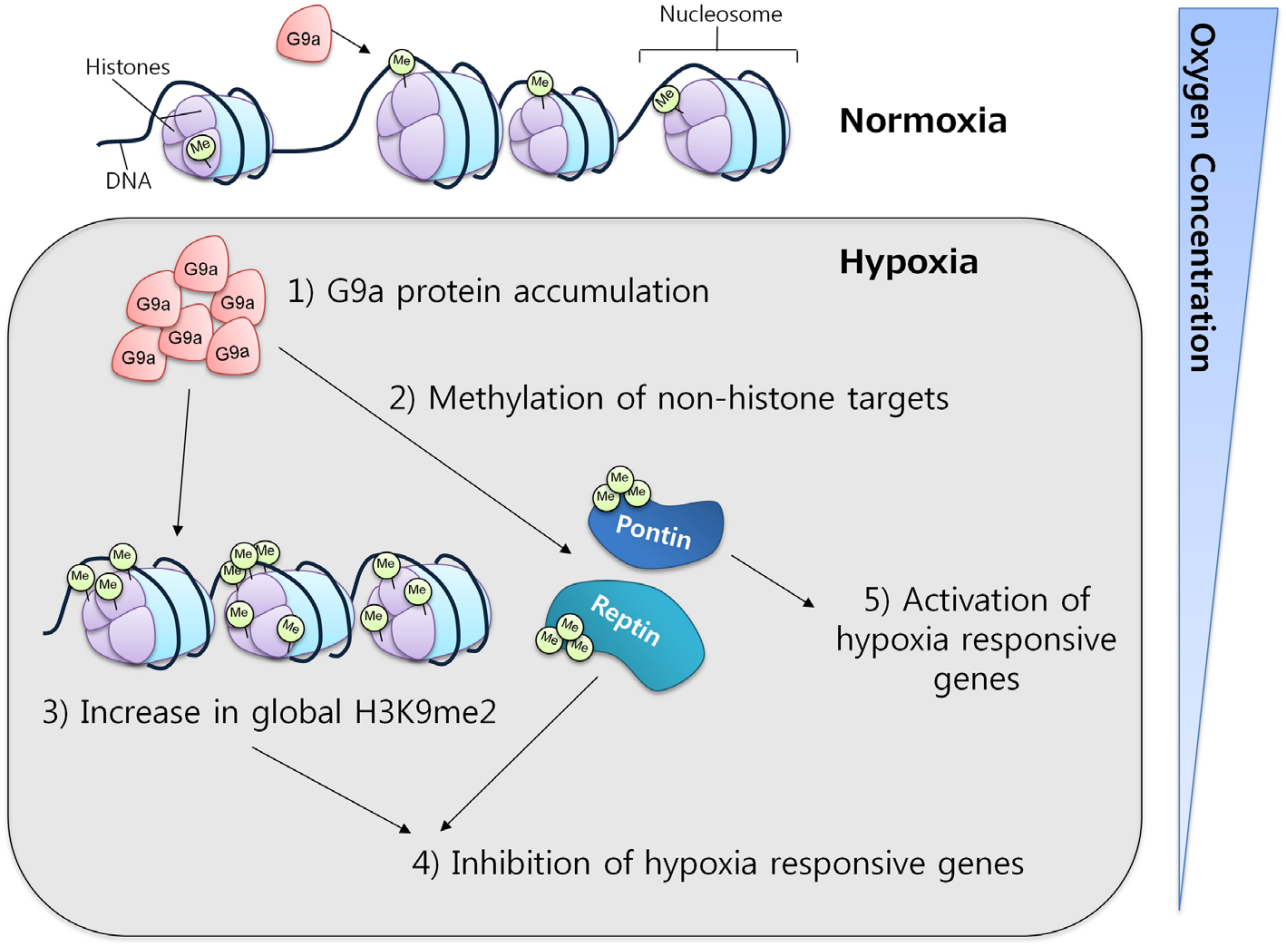
**G9a in hypoxia**. G9a activity is enhanced under hypoxic conditions, leading to the repression of a specific subset of hypoxia-responsive genes. Under similar conditions, G9a can also methylate non-histone proteins, such as Pontin and Reptin, respectively activating or inhibiting the expression of various target genes.

In mammalian cell lines, G9a activity has been observed to increase under hypoxic conditions through protein accumulation. This increase was correlated with a concomitant increase in the global levels of histone H3K9 di-methylation, which in turn resulted in gene silencing, providing evidence for a critical function of G9a in the repression of genes in response to hypoxia (Figure 4) (61, 62). The ability of G9a to actively repress genes in hypoxic conditions suggests a key role in the promotion of cell survival under this condition. In solid tumors, where hypoxia is a common micro-environmental state, G9a might be functioning as a factor that enhances survival, proliferation, and metastasis of malignant cells.

### Non-histone targets of G9a

Apart from histones, G9a has also been found to methylate other proteins. While the implications of this mechanism are not fully understood, it is clear that G9a may exert important functions through this pathway. It is known that non-histonic methylation by G9a can have either a repressive or an activator effect on gene expression. For instance, during hypoxia, Reptin, a chromatin-remodeling factor, was found to be methylated at K67 by G9a. This induced Reptin to recruit HDAC1 to hypoxia-responsive gene promoters attenuating the transcriptional activity of HIF-1α (63). In contrast, under similar conditions, G9a can also methylate another chromatin-remodeling factor, Pontin, enhancing the transcriptional activity of HIF-1α by recruiting p300/CBP to a subset of hypoxia-responsive genes (64). These results not only demonstrate the activity of G9a toward non-histones but also strengthen the evidence for its importance in mediating the hypoxia-response.

Another G9a non-histone target is the tumor suppressor p53, a sequence-specific transcription factor, known to be mutated in a substantial proportion of human tumors. Transcriptional activation and repression of p53 was observed to be mediated through various mechanisms, including protein methylation. G9a is indeed responsible for p53 methylation at lysine 373, and this form of the transcription factor was observed to be inactive (65). During skeletal muscle differentiation, myogenic regulatory factors such as MyoD play a key role. This factor regulates gene expression and it is one of the genes responsible for skeletal muscle development. G9a is able to regulate MyoD activity by specifically methylating K104. Methylated MyoD is inactive, while the methylation-defective mutants were able to actively promote differentiation (66).

There are several other proteins identified to be G9a targets including CDYL1, WIZ, and ACINUS (67). Methylation of CDYL1 was found to alter its chromodomain binding to H3K9me3 suggesting that it may regulate the activity of chromatin factors and interaction. Interestingly, G9a was also observed to undergo automethylation. G9a is automethylated at K239, facilitating the binding of HP1 at the methylated site (68). While its biological significance remains elusive, it is possible that this process is required for the recruitment of other factors. In fact, it is known that HP1 actively interacts with DNMT1 and that the recruitment of these factors, together with G9a, coincides with gene silencing (69). DNMT1 itself was also observed to be methylated by another HMT, SET-7, specifically at K142. During the S and G2 phase of the cell cycle, the amount of methylated DNMT1 increases, leading to its degradation (70). These observations increase the evidence for the existence of a tight crosstalk between DNA and histone modifications, demonstrating that one can have a strong influence in the regulation of the other.

## G9a in Cancer

### Deregulated levels of G9a in various tumor types

While cancer has commonly been considered as a disease, which mainly arises from the accumulation of genetic mutations, it is now understood that alterations in the modification of both DNA and histones (the epigenome) contribute to the initiation and progression of cancer. In fact, cancer cells are characterized by clear epigenetic misregulations, which have been observed in a variety of cancers, including breast, lung, head and neck, brain, and ovarian carcinoma (2, 71–75). In this topic, G9a has attracted particular attention for its role in the promotion of tumorigenesis.

**Figure 5:**
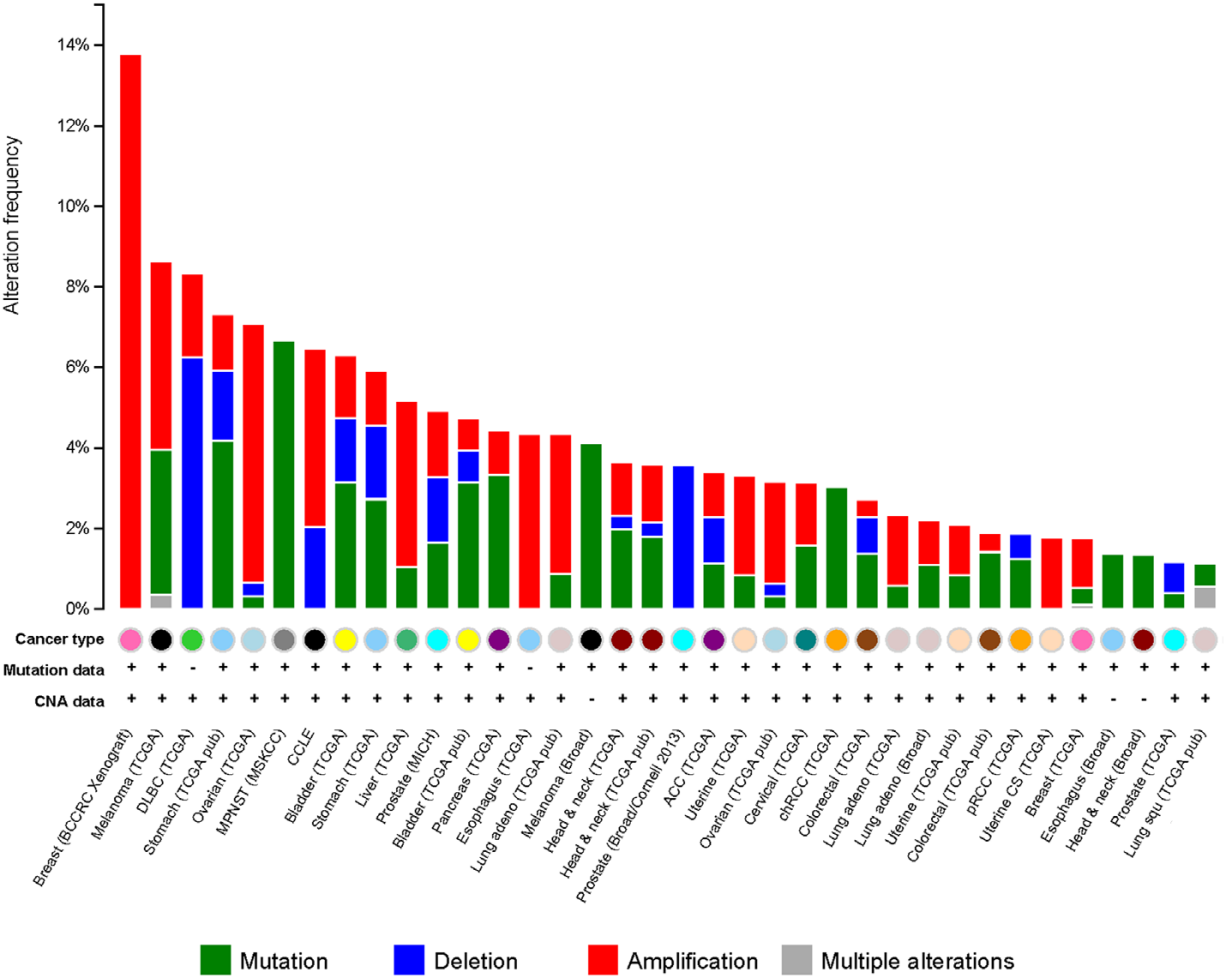
**G9a alterations in cancer**. Genetic alterations for G9a in different types of cancer from cBioportal (www.cbioportal.org). Genetic alterations are shown as green (mutations), blue (deletions), red (amplifications), and gray (multiple alterations).

It has been observed that G9a is overexpressed in a number of cancers, including esophageal squamous cell carcinoma, hepatocellular carcinoma, aggressive lung cancer, brain cancer, multiple myeloma, and aggressive ovarian carcinoma (Figure 5) (76–79). Higher G9a expression levels were also noted to be associated with poor prognosis (2, 65, 74, 79). Elevated G9a levels were commonly correlated with higher methylation levels, leading to the suppression of important tumor suppressor genes. In breast cancer, the metastasis suppressor genes desmocollin 3 (DSC3) and MASPIN, for instance, were reported to be frequently silenced by an epigenetic mechanism (80). DSC3 is a glycoprotein, belonging to the cadherin superfamily, required for the desmosome-mediated cell-to-cell junction and adhesion (81). MASPIN is a protease inhibitor, which was shown to reduce the ability to induce tumor growth and metastasis (82). Pharmacologic inhibition of G9a has been demonstrated using the DNA methyltransferase inhibitor 5-Aza-2′-deoxycytidine, as well as RNA interference (RNAi)-mediated silencing. Inhibition of G9a led to the reactivation of the two tumor suppressors concomitantly, with a reduction of H3K9 di-methylation mark (80) suggesting that the activity of G9a may be linked to DNA methylation. A runt-domain transcription factor, RUNX3 is known to act as a tumor suppressor gene in gastric cancer, and its level of expression was dependent on post-translational modifications, such as acetylation and sumoylation. Transcriptional repression of this factor was instead observed to be mediated by methylation with G9a found to be responsible for its hypoxia-mediated silencing, increasing H3K9 di-methylation and decreasing H3 acetylation at the promoter region of the gene (83). In addition, in ovarian cancer, G9a activity promoted the suppression of different tumor suppressors, including *CDH1*, *DUSP5*, *SPRY4*, and *PPP1R15A* (79). Moreover, as previously mentioned, G9a methylates the tumor suppressor p53, leading to its inactivation (65). It is thus believed that targeting G9a in cancer will lead to the re-expression of important tumor suppressor genes.

Overexpression of G9a, and not of its related protein GLP, has often been associated with a more aggressive phenotype in cancer. For instance, elevated G9a protein levels were observed in the highly invasive lung cancer cell lines CL1-5 and H1299 as a result of gene amplification. In contrast, poorly invasive CL1-0 cells are characterized by a lower expression of the enzyme (2). Even though the global H3K9me2 levels were not directly correlated with G9a protein expression levels, ectopic overexpression of the enzyme in CL1-0 increased cell motility and invasiveness, whereas its silencing in fast-growing cell lines led to a less aggressive phenotype (2). Similar results were also observed in ovarian cancer, where the invasive cell lines ES-2, SKOV-3, TOV-21G, OV-90, and OVCAR-3 were found to be characterized by elevated G9a levels compared with poorly aggressive tumor cells (79). In addition, G9a protein levels were also found to be significantly correlated with the disease stage in ovarian cancer, with a lower and higher expression of the enzyme found to characterize early and late phase of the disease, respectively (79). This data suggest that G9a expression levels influence cell motility in cancer in a target-specific manner by altering histone H3K9 methylation status. Moreover, ovarian cancer xenografts have demonstrated a higher expression of the methyltransferase in metastatic lesions compared to the primary tumors, whereas knocked down G9a was able to reduce metastasis *in vivo* in lung cancer, demonstrating a direct association between G9a protein levels and metastasis (79).

It is clear that an appropriate level of G9a activity is required to maintain the normal phenotype in a cell. During hypoxia, G9a activity increases, causing an increase in global histone methylation. As hypoxia is considered to be an important factor in the development of metastasis of solid tumors, the acquisition of cell motility under hypoxic conditions has been correlated with a reduction in the expression of cell adhesion molecules (84). In this context, G9a was demonstrated to inhibit the expression of cell adhesion factors such as E-cadherin and epithelial cell adhesion molecules (Ep-cam). Inhibition of G9a led to the re-expression of these molecules and to a reduction in motility and metastasis *in vivo* in aggressive lung and breast cancer (2, 72). The correlation between G9a-mediated repression of cell adhesion molecules and its increase in activity during hypoxia strongly supports the evidence for a direct involvement of G9a in the metastatic pathway. This hypothesis is also supported by the fact that overexpression of G9a was often observed in aggressive and highly metastatic forms of cancer, indicating that G9a expression might be a key factor in the occurrence of metastasis (2, 79).

These findings demonstrate the importance of G9a in the maintenance of the malignant phenotype and suggest that targeting this enzyme might represent a novel strategy for the treatment of various types of solid tumors, characterized by hypoxic regions and higher risk of metastasis.

### G9a inhibition and effects on cell proliferation

G9a depletion has been reported to inhibit cell proliferation in several cancer cell lines (75, 85, 86). In cancer, an increased activation of the serine–glycine biosynthetic pathway is commonly observed, which is known to drive the synthesis of macromolecules fundamental for cell proliferation and promoting cancer cell survival. There are many reports demonstrating the requirement of G9a for maintaining an active pathway through H3K9 mono-methylation (87). Interestingly, various studies have shown that G9a inhibition ultimately leads to autophagy. Autophagy is a cell survival mechanism in which proteins and organelles are degraded in response to cellular stress, such as hypoxic conditions or nutrient deprivation. Fundamental for the autophagic response is the inhibition of mTOR, a signaling pathway able to sense environmental conditions and regulate growth (75, 88). Silencing or blocking G9a function was sufficient in inhibiting mTOR, leading to autophagic cell death in head and neck squamous carcinoma (75).

The addition of serine to the cell culture medium was able to rescue G9a-inhibited cells from autophagy, demonstrating a defect in the serine metabolic pathway induced by depletion of G9a. Together, these data suggest that targeting G9a may lead to a reduction in cancer proliferation caused by autophagic death through imbalance in the serine–glycine biosynthetic pathway.

### Pharmacological inhibition of G9a

There is a growing evidence of a critical role for G9a in the initiation and progression of solid tumors. This led to the hypothesis that targeting G9a and its epigenetic machinery would promote the re-expression of tumor suppressor genes, a reduction in metastasis and the inhibition of cancer cell proliferation. A number of small molecule inhibitors have been developed with the capacity to inhibit G9a catalytic activity and have been used in various *in vitro* and *in vivo* experiments. One of the first molecules developed was BIX-01294 (diazepin-quinazolin-amine derivative), a competitive inhibitor specific for G9a, able to reduce G9a-mediated H3K9 di-methylation, but not mono-methylation (89). BIX-01294 competes with G9a substrate and not with G9a cofactor *S*-adenosyl-methionine (SAM), the source of the transferred methyl group (89, 90). BIX-01294 treatment was shown to reduce cell proliferation in leukemia HL-60 and NB4 cell lines, as well as in human germ cell tumors and squamous neck carcinoma (91, 92). Moreover, cells pre-treated with the inhibitor and subsequently injected into mice formed significantly smaller tumors when compared with untreated ones, suggesting that G9a inhibition could effectively reduce tumor growth and metastatic potential (92). However, BIX-01294 also showed intrinsic toxic effects, which were not related to the inhibition of G9a-mediated methylation. The molecule has then been optimized, leading to the synthesis of a second inhibitor, UNC0638, which exhibited high potency and specificity for G9a, combined with lower cell toxicity, as well as higher lipophilic characteristics and cell membrane permeability (93). UNC0638 has efficiently been used *in vitro*, suppressing cellular proliferation in various cancer cell lines, such as breast, squamous head and neck carcinoma, hepatocellular carcinoma, acute myeloid leukemia, and cervical cancer (58, 92–94). However, while RNAi and BIX-01294-mediated inhibition of G9a was shown to induce autophagy (95) (through mTOR inhibition, as previously discussed), pharmacologic inhibition of the enzyme using UNC0638 did not lead to the same phenotype (75, 94). In particular, mTOR appeared not to be inhibited following UNC0638 treatment. This contrasting evidence might be due to the different mTOR targets compared in the two experiments, or by the use of different cell lines. It is nevertheless possible that the dissimilar response is due to the use of different inhibitors, suggesting that these inhibitors might in some way display diverse effects.

Although UNC0638 displays improved chemical characteristics with respect to previous inhibitors, it is affected by a poor pharmacokinetics, which impedes its efficient use *in vivo*. Recently, Liu and its group reported the synthesis of the G9a and GLP inhibitor suitable for animal studies, UNC0642 (96). The molecule was shown to demonstrate an improved pharmacokinetics, while it maintained a high selectivity and low cell toxicity. Thus, UNC0642 represents a promising candidate for targeting the enzyme in animal models, which will allow the evaluation of G9a functions in different cancer setting.

### Inhibitors to other epigenetic modifying enzymes

A growing number of epigenetic inhibitors has been developed and tested to specifically inhibit dysregulated enzymes in a broad array of human diseases. Epigenetic drugs have the potential to reverse the adverse effects acquired from genetic mutations with the advantage of being less invasive if compared with other new technologies, such as gene therapies. However, inhibitors lack target selectivity, and may cause cell type-specific changes in gene expression, which can eventually lead to disruption of various cellular pathways and non-specific cell death (97, 98).

Epigenetic drugs mainly comprehend DNA methyltransferases inhibitors, and some of these molecules are now being tested in clinical trials. In particular, 5-azacytidine (Vidaza) has received the FDA approval for the treatment of myelodysplastic syndromes (99). However, other epigenetic drugs, which target histone modifiers have also been developed, which mainly comprehend HDAC inhibitors and lysine methyltransferase inhibitors (Table 1). While HMT inhibitors have not yet reached the clinical trial stage, various HDAC inhibitors have been approved by the FDA and are undergoing trials in different types of malignancies, such as ovarian carcinoma and leukemia (100, 101). Vorinostat is probably the most advanced HDAC inhibitor to date, approved by the FDA in 2006, demonstrating that it is well tolerated and have promising anticancer activity in combination with other chemotherapeutic drugs (102). In fact, combinatorial therapy using epigenetic inhibitors together with other drugs, like chemotherapy and hormonal therapy, has the ability to improve the efficacy of existing treatments. For instance, epigenetic drugs are expected to possibly sensitize resistant cells to their present therapies, through the re-expression of fundamental genes, such as important tumor suppressors (103). Thus, epigenetic enzymes represent key target molecules for various kinds of diseases affected by deregulations in gene expression patterns. Substantial progress has been made in the understanding of their roles in human pathologies and in the development of small molecule inhibitors. New technologies are nevertheless needed to increase their specificity in order to obtain an intelligent control of gene expression.

**Table 1:** **Epigenetic enzymes and available inhibitors**.

Enzyme	Inhibitor
**Histone Lysine Methyltransferase**	
Dot1l/KMT 4	EPZ004777, EPZ005676 (104)
KMT6	3-deazaneplanocin A (DZNep) (105)
G9a/GLP	BIX (84), UNC0638 (93), UNC0642 (96)
SUV39H1	Chaetocin (106)
SUV39H2	Chaetocin (106)
EZH2	GSK343, GSK126, EPZ006438, EPZ005687 (99)
**Histone Lysine Demethylases**	
KDM2/7	Daminozide (107), TC-E 5002 (108)
JMJD2A	DMOG (109)
KDM6B and KDM6A combined	GSK-J1/J4 (110)
JMJC family	IOX1 (111)
JMJD2	ML324 (112)
LSD1	PCPA (phenylcyclopropylamine) (113)
Novel histone demethylase LSD1 inhibitors	RN-1 (113), TCP (114), CAS 927019-63-4 (115), CBB1007 (116), S2101 (116)
Jumonji family	JIB-04 (117)
**Histone Acetyltransferases**	
GCN5/KAT2A	MB3 (butyrolactone 3) (118)
PCAF/KAT3A + p300/KAT3B	EML425 (119)
PCAF/KAT3A	H3COA20 (120)
p300/KAT3B	Curcumin (121), LTK 14 (122), LYS-COA (120)
p300/KAT3B + CBP/KAT3A	C646 (123), histone acetyltransferase inhibitor II (124)
p300/KAT3B + PCAF/KAT2B	Garcinol (125)
**Histone Deacetylases**	
Class I (HDAC1, HDAC2, HDAC3, HDAC8, HDAC4)	Pyroxamide (126), tacedinaline (127), mocetinostat (GCD 0103) (128)
Class I/II (HDAC1, HDAC2, HDAC3, HDAC8, HDAC4, HDAC5, HDAC6, HDAC7, HDAC9, HDAC10)	Vorinostat (SAHA) (129), belinostat (130), LAQ 824 (131), panobinostat (130), givinostat (130), abexinostat (PCI 24781) (132), sodium phenylbutyrate (133), valproic acid, trichostatin A (134)
HDAC1, HDAC2, HDAC3	Entinostat (135)
HDAC1, HDAC2	Romidepsin (130)
Unknown specificity	Pivanex (AN-9) (136)

## Conclusions and Future Directions

Recent findings have suggested an important role for G9a in the progression of solid tumors, promoting cell proliferation and survival under hypoxic conditions, as well as metastasis. Further studies focusing on determining the transcriptional role of G9a in various cancer types will provide better understanding of epigenetic changes that occur in cancer. To fully understand the intricacies of G9a’s contribution to gene regulation in cancer, future work utilizing small molecule inhibitors to G9a both *in vitro* and *in vivo* are required to determine whether inhibition of G9a is beneficial at a physiological setting. Characterization of G9a target genes by whole transcriptome analysis and the resultant changes in the histone methylation status monitored by chromatin immunoprecipitation (ChIP) in the setting of tumor hypoxia and pharmacologic inhibition will play a central role in assessing the therapeutic value. It appears that G9a activity is fundamental for the maintenance of the malignant phenotype in several cancer types and modulating the expression of genes regulated by G9a may have the potential of developing more effective treatment. The G9a inhibitor can be used alone or in combination with other standard-of care therapies currently used in the clinic in order to develop better treatments for cancer patients, including the aggressive metastatic subtypes for which no efficient treatment is available.

## Author Contributions

KW generated the figures. FG had contributed to the design of the work, FC and JL had substantial contributions to the conception and design of the work, drafted and revised the manuscript, had final approval of the version to be published and agree to be accountable for all aspects of the work.

## Conflict of Interest Statement

The authors declare that the research was conducted in the absence of any commercial or financial relationships that could be construed as a potential conflict of interest.

## Abbreviations

CAM, cell adhesion molecule; ChIP, chromatin immunoprecipitation; DSC3, desmocollin 3; Ep-cam, epithelial cell adhesion molecules; ES, embryonic stem cells; GLP, G9a-like protein; H2A, histone variant 2A; H2B, histone variant 2B; H2BK123, histone variant 2B lysine 123; H3K4, histone 3 lysine 4; H3K9, histone 3 lysine 9; H3K14, histone 3 lysine 14; H3K79, histone 3 lysine 79; H3S10, histone 3 serine 10; HAT, histone acetyltransferase; HDAC, histone deacetyltransferase; HDM, histone demethylase; HMT, histone methyltransferase; HP1, heterochromatin protein 1; JmjC, Jumonji C domain; KMTs, lysine methyltransferase; LSD1, lysine-specific demethylase 1; RNAi, RNA interfering; SET, Su(var) 3-9, enhancer of zeste (Ez) and trithorax homologous domain; SUMO, small ubiquitin-related modifier.

## References

[1] Baxter E Windloch K Gannon F Lee JS. Epigenetic regulation in cancer progression. Cell Biosci (2014) 4:45. 10.1186/2045-3701-4-45 25949794 PMC4422217

[2] Chen MW Hua KT Kao HJ Chi CC Wei LH Johansson G H3K9 histone methyltransferase G9a promotes lung cancer invasion and metastasis by silencing the cell adhesion molecule Ep-CAM. Cancer Res (2010) 70(20):7830–40. 10.1158/0008-5472.CAN-10-0833 20940408

[3] Egger G Liang G Aparicio A Jones PA. Epigenetics in human disease and prospects for epigenetic therapy. Nature (2004) 429(6990):457–63. 10.1038/nature02625 15164071

[4] Waddington CH Towards a theoretical biology. Nature (1968) 218(5141):525–7. 10.1038/218525a0 5650959

[5] Luger K Rechsteiner TJ Flaus AJ Waye MM Richmond TJ. Characterization of nucleosome core particles containing histone proteins made in bacteria. J Mol Biol (1997) 272(3):301–11. 10.1006/jmbi.1997.1235 9325091

[6] Shiio Y Eisenman RN. Histone sumoylation is associated with transcriptional repression. Proc Natl Acad Sci U S A (2003) 100(23):13225–30. 10.1073/pnas.1735528100 14578449 PMC263760

[7] Biran A Meshorer E. Concise review: chromatin and genome organization in reprogramming. Stem Cells (2012) 30(9):1793–9. 10.1002/stem.1169 22782851

[8] Jenuwein T Allis CD. Translating the histone code. Science (2001) 293(5532):1074–80. 10.1126/science.1063127 11498575

[9] Hong L Schroth GP Matthews HR Yau P Bradbury EM. Studies of the DNA binding properties of histone H4 amino terminus. Thermal denaturation studies reveal that acetylation markedly reduces the binding constant of the H4 “tail” to DNA. J Biol Chem (1993) 268(1):305–14. 8416938

[10] Lee DY Hayes JJ Pruss D Wolffe AP. A positive role for histone acetylation in transcription factor access to nucleosomal DNA. Cell (1993) 72(1):73–84. 10.1016/0092-8674(93)90051-Q 8422685

[11] Wade PA Pruss D Wolffe AP. Histone acetylation: chromatin in action. Trends Biochem Sci (1997) 22(4):128–32. 10.1016/S0968-0004(97)01016-5 9149532

[12] Huang X King MA Halicka HD Traganos F Okafuji M Darzynkiewicz Z. Histone H2AX phosphorylation induced by selective photolysis of BrdU-labeled DNA with UV light: relation to cell cycle phase. Cytometry A (2004) 62(1):1–7. 10.1002/cyto.a.20086 15455410

[13] Stewart GS Wang B Bignell CR Taylor AM Elledge SJ. MDC1 is a mediator of the mammalian DNA damage checkpoint. Nature (2003) 421(6926):961–6. 10.1038/nature01446 12607005

[14] Stucki M Clapperton JA Mohammad D Yaffe MB Smerdon SJ Jackson SP. MDC1 directly binds phosphorylated histone H2AX to regulate cellular responses to DNA double-strand breaks. Cell (2005) 123(7):1213–26. 10.1016/j.cell.2005.09.038 16377563

[15] Tsukada Y Fang J Erdjument-Bromage H Warren ME Borchers CH Tempst P Histone demethylation by a family of JmjC domain-containing proteins. Nature (2006) 439(7078):811–6. 10.1038/nature04433 16362057

[16] Barratt MJ Hazzalin CA Cano E Mahadevan LC. Mitogen-stimulated phosphorylation of histone H3 is targeted to a small hyperacetylation-sensitive fraction. Proc Natl Acad Sci U S A (1994) 91(11):4781–5. 10.1073/pnas.91.11.4781 8197135 PMC43872

[17] Metzger E Imhof A Patel D Kahl P Hoffmeyer K Friedrichs N Phosphorylation of histone H3T6 by PKCbeta(I) controls demethylation at histone H3K4. Nature (2010) 464(7289):792–6. 10.1038/nature08839 20228790

[18] Anest V Hanson JL Cogswell PC Steinbrecher KA Strahl BD Baldwin AS. A nucleosomal function for IkappaB kinase-alpha in NF-kappaB-dependent gene expression. Nature (2003) 423(6940):659–63. 10.1038/nature01648 12789343

[19] Dawson MA Bannister AJ Gottgens B Foster SD Bartke T Green AR JAK2 phosphorylates histone H3Y41 and excludes HP1alpha from chromatin. Nature (2009) 461(7265):819–22. 10.1038/nature08448 19783980 PMC3785147

[20] Henry KW Wyce A Lo WS Duggan LJ Emre NC Kao CF Transcriptional activation via sequential histone H2B ubiquitylation and deubiquitylation, mediated by SAGA-associated Ubp8. Genes Dev (2003) 17(21):2648–63. 10.1101/gad.1144003 14563679 PMC280615

[21] Nathan D Ingvarsdottir K Sterner DE Bylebyl GR Dokmanovic M Dorsey JA Histone sumoylation is a negative regulator in *Saccharomyces cerevisiae* and shows dynamic interplay with positive-acting histone modifications. Genes Dev (2006) 20(8):966–76. 10.1101/gad.1404206 16598039 PMC1472304

[22] Dhall A Wei S Fierz B Woodcock CL Lee TH Chatterjee C. Sumoylated human histone H4 prevents chromatin compaction by inhibiting long-range internucleosomal interactions. J Biol Chem (2014) 289(49):33827–37. 10.1074/jbc.M114.591644 25294883 PMC4256319

[23] Tachibana M Matsumura Y Fukuda M Kimura H Shinkai Y. G9a/GLP complexes independently mediate H3K9 and DNA methylation to silence transcription. EMBO J (2008) 27(20):2681–90. 10.1038/emboj.2008.192 18818694 PMC2572175

[24] Shi X Kachirskaia I Walter KL Kuo JH Lake A Davrazou F Proteome-wide analysis in *Saccharomyces cerevisiae* identifies several PHD fingers as novel direct and selective binding modules of histone H3 methylated at either lysine 4 or lysine 36. J Biol Chem (2007) 282(4):2450–5. 10.1074/jbc.C600286200 17142463 PMC2735445

[25] Barski A Cuddapah S Cui K Roh TY Schones DE Wang Z High-resolution profiling of histone methylations in the human genome. Cell (2007) 129(4):823–37. 10.1016/j.cell.2007.05.009 17512414

[26] Shilatifard A. Chromatin modifications by methylation and ubiquitination: implications in the regulation of gene expression. Annu Rev Biochem (2006) 75:243–69. 10.1146/annurev.biochem.75.103004.142422 16756492

[27] Martin C Zhang Y. The diverse functions of histone lysine methylation. Nat Rev Mol Cell Biol (2005) 6(11):838–49. 10.1038/nrm1761 16261189

[28] Black JC Van Rechem C Whetstine JR. Histone lysine methylation dynamics: establishment, regulation, and biological impact. Mol Cell (2012) 48(4):491–507. 10.1016/j.molcel.2012.11.006 23200123 PMC3861058

[29] Sun XJ Wei J Wu XY Hu M Wang L Wang HH Identification and characterization of a novel human histone H3 lysine 36-specific methyltransferase. J Biol Chem (2005) 280(42):35261–71. 10.1074/jbc.M504012200 16118227

[30] Kirmizis A Santos-Rosa H Penkett CJ Singer MA Vermeulen M Mann M Arginine methylation at histone H3R2 controls deposition of H3K4 trimethylation. Nature (2007) 449(7164):928–32. 10.1038/nature06160 17898715 PMC3350864

[31] Bauer UM Daujat S Nielsen SJ Nightingale K Kouzarides T. Methylation at arginine 17 of histone H3 is linked to gene activation. EMBO Rep (2002) 3(1):39–44. 10.1093/embo-reports/kvf013 11751582 PMC1083932

[32] Zhao Q Rank G Tan YT Li H Moritz RL Simpson RJ PRMT5-mediated methylation of histone H4R3 recruits DNMT3A, coupling histone and DNA methylation in gene silencing. Nat Struct Mol Biol (2009) 16(3):304–11. 10.1038/nsmb.1568 19234465 PMC5120857

[33] Rea S Eisenhaber F O’Carroll D Strahl BD Sun ZW Schmid M Regulation of chromatin structure by site-specific histone H3 methyltransferases. Nature (2000) 406(6796):593–9. 10.1038/35020506 10949293

[34] Wassenegger M Heimes S Riedel L Sanger HL. RNA-directed de novo methylation of genomic sequences in plants. Cell (1994) 76(3):567–76. 10.1016/0092-8674(94)90119-8 8313476

[35] Yang Y Bedford MT. Protein arginine methyltransferases and cancer. Nat Rev Cancer (2013) 13(1):37–50. 10.1038/nrc3409 23235912

[36] Santos-Rosa H Schneider R Bannister AJ Sherriff J Bernstein BE Emre NC Active genes are tri-methylated at K4 of histone H3. Nature (2002) 419(6905):407–11. 10.1038/nature01080 12353038

[37] Nielsen SJ Schneider R Bauer UM Bannister AJ Morrison A O’Carroll D Rb targets histone H3 methylation and HP1 to promoters. Nature (2001) 412(6846):561–5. 10.1038/35087620 11484059

[38] Wysocka J Swigut T Milne TA Dou Y Zhang X Burlingame AL WDR5 associates with histone H3 methylated at K4 and is essential for H3 K4 methylation and vertebrate development. Cell (2005) 121(6):859–72. 10.1016/j.cell.2005.03.036 15960974

[39] Wang Y Wysocka J Sayegh J Lee YH Perlin JR Leonelli L Human PAD4 regulates histone arginine methylation levels via demethylimination. Science (2004) 306(5694):279–83. 10.1126/science.1101400 15345777

[40] Shi Y Lan F Matson C Mulligan P Whetstine JR Cole PA Histone demethylation mediated by the nuclear amine oxidase homolog LSD1. Cell (2004) 119(7):941–53. 10.1016/j.cell.2004.12.012 15620353

[41] Lewis A Mitsuya K Umlauf D Smith P Dean W Walter J Imprinting on distal chromosome 7 in the placenta involves repressive histone methylation independent of DNA methylation. Nat Genet (2004) 36(12):1291–5. 10.1038/ng1468 15516931

[42] Campbell MJ Turner BM Altered histone modifications in cancer. Adv Exp Med Biol (2013) 754:81–107. 10.1007/978-1-4419-9967-2_4 22956497

[43] Bannister AJ Kouzarides T. Regulation of chromatin by histone modifications. Cell Res (2011) 21(3):381–95. 10.1038/cr.2011.22 21321607 PMC3193420

[44] Strahl BD Allis CD. The language of covalent histone modifications. Nature (2000) 403(6765):41–5. 10.1038/47412 10638745

[45] Lo WS Trievel RC Rojas JR Duggan L Hsu JY Allis CD Phosphorylation of serine 10 in histone H3 is functionally linked in vitro and in vivo to Gcn5-mediated acetylation at lysine 14. Mol Cell (2000) 5(6):917–26. 10.1016/S1097-2765(00)80257-9 10911986

[46] Shahbazian MD Zhang K Grunstein M. Histone H2B ubiquitylation controls processive methylation but not monomethylation by Dot1 and Set1. Mol Cell (2005) 19(2):271–7. 10.1016/j.molcel.2005.06.010 16039595

[47] Nakanishi S Sanderson BW Delventhal KM Bradford WD Staehling-Hampton K Shilatifard A. A comprehensive library of histone mutants identifies nucleosomal residues required for H3K4 methylation. Nat Struct Mol Biol (2008) 15(8):881–8. 10.1038/nsmb.1454 18622391 PMC2562305

[48] Esteve PO Chin HG Smallwood A Feehery GR Gangisetty O Karpf AR Direct interaction between DNMT1 and G9a coordinates DNA and histone methylation during replication. Genes Dev (2006) 20(22):3089–103. 10.1101/gad.1463706 17085482 PMC1635145

[49] Milner CM Campbell RD. The G9a gene in the human major histocompatibility complex encodes a novel protein containing ankyrin-like repeats. Biochem J (1993) 290(Pt 3):811–8. 10.1042/bj2900811 8457211 PMC1132354

[50] Collins R Cheng X. A case study in cross-talk: the histone lysine methyltransferases G9a and GLP. Nucleic Acids Res (2010) 38(11):3503–11. 10.1093/nar/gkq081 20159995 PMC2887955

[51] Tachibana M Ueda J Fukuda M Takeda N Ohta T Iwanari H Histone methyltransferases G9a and GLP form heteromeric complexes and are both crucial for methylation of euchromatin at H3-K9. Genes Dev (2005) 19(7):815–26. 10.1101/gad.1284005 15774718 PMC1074319

[52] Lachner M O’Carroll D Rea S Mechtler K Jenuwein T. Methylation of histone H3 lysine 9 creates a binding site for HP1 proteins. Nature (2001) 410(6824):116–20. 10.1038/35065132 11242053

[53] Peters AH O’Carroll D Scherthan H Mechtler K Sauer S Schofer C Loss of the Suv39h histone methyltransferases impairs mammalian heterochromatin and genome stability. Cell (2001) 107(3):323–37. 10.1016/S0092-8674(01)00542-6 11701123

[54] Rice JC Briggs SD Ueberheide B Barber CM Shabanowitz J Hunt DF Histone methyltransferases direct different degrees of methylation to define distinct chromatin domains. Mol Cell (2003) 12(6):1591–8. 10.1016/S1097-2765(03)00479-9 14690610

[55] Tachibana M Sugimoto K Nozaki M Ueda J Ohta T Ohki M G9a histone methyltransferase plays a dominant role in euchromatic histone H3 lysine 9 methylation and is essential for early embryogenesis. Genes Dev (2002) 16(14):1779–91. 10.1101/gad.989402 12130538 PMC186403

[56] Feldman N Gerson A Fang J Li E Zhang Y Shinkai Y G9a-mediated irreversible epigenetic inactivation of Oct-3/4 during early embryogenesis. Nat Cell Biol (2006) 8(2):188–94. 10.1038/ncb1353 16415856

[57] Antignano F Burrows K Hughes MR Han JM Kron KJ Penrod NM Methyltransferase G9A regulates T cell differentiation during murine intestinal inflammation. J Clin Invest (2014) 124(5):1945–55. 10.1172/JCI69592 24667637 PMC4001530

[58] Lehnertz B Northrop JP Antignano F Burrows K Hadidi S Mullaly SC Activating and inhibitory functions for the histone lysine methyltransferase G9a in T helper cell differentiation and function. J Exp Med (2010) 207(5):915–22. 10.1084/jem.20100363 20421388 PMC2867284

[59] Osipovich O Milley R Meade A Tachibana M Shinkai Y Krangel MS Targeted inhibition of V(D)J recombination by a histone methyltransferase. Nat Immunol (2004) 5(3):309–16. 10.1038/ni1042 14985714

[60] Thomas LR Miyashita H Cobb RM Pierce S Tachibana M Hobeika E Functional analysis of histone methyltransferase g9a in B and T lymphocytes. J Immunol (2008) 181(1):485–93. 10.4049/jimmunol.181.1.485 18566414 PMC2497432

[61] Chen H Yan Y Davidson TL Shinkai Y Costa M. Hypoxic stress induces dimethylated histone H3 lysine 9 through histone methyltransferase G9a in mammalian cells. Cancer Res (2006) 66(18):9009–16. 10.1158/0008-5472.CAN-06-0101 16982742

[62] Wang J Lu F Ren Q Sun H Xu Z Lan R Novel histone demethylase LSD1 inhibitors selectively target cancer cells with pluripotent stem cell properties. Cancer Res (2011) 71(23):7238–49. 10.1158/0008-5472.CAN-11-0896 21975933 PMC3228901

[63] Lee JS Kim Y Kim IS Kim B Choi HJ Lee JM Negative regulation of hypoxic responses via induced reptin methylation. Mol Cell (2010) 39(1):71–85. 10.1016/j.molcel.2010.06.008 20603076 PMC4651011

[64] Lee JS Kim Y Bhin J Shin HJ Nam HJ Lee SH Hypoxia-induced methylation of a pontin chromatin remodeling factor. Proc Natl Acad Sci U S A (2011) 108(33):13510–5. 10.1073/pnas.1106106108 21825155 PMC3158161

[65] Huang J Dorsey J Chuikov S Perez-Burgos L Zhang X Jenuwein T G9a and Glp methylate lysine 373 in the tumor suppressor p53. J Biol Chem (2010) 285(13):9636–41. 10.1074/jbc.M109.062588 20118233 PMC2843213

[66] Jin W Liu Y Chen L Zhu H Di GH Ling H Involvement of MyoD and c-myb in regulation of basal and estrogen-induced transcription activity of the BRCA1 gene. Breast Cancer Res Treat (2011) 125(3):699–713. 10.1007/s10549-010-0876-1 20364308

[67] Rathert P Dhayalan A Murakami M Zhang X Tamas R Jurkowska R Protein lysine methyltransferase G9a acts on non-histone targets. Nat Chem Biol (2008) 4(6):344–6. 10.1038/nchembio.88 18438403 PMC2696268

[68] Chin HG Esteve PO Pradhan M Benner J Patnaik D Carey MF Automethylation of G9a and its implication in wider substrate specificity and HP1 binding. Nucleic Acids Res (2007) 35(21):7313–23. 10.1093/nar/gkm726 17962312 PMC2175347

[69] Smallwood A Esteve PO Pradhan S Carey M. Functional cooperation between HP1 and DNMT1 mediates gene silencing. Genes Dev (2007) 21(10):1169–78. 10.1101/gad.1536807 17470536 PMC1865489

[70] Esteve PO Chin HG Benner J Feehery GR Samaranayake M Horwitz GA Regulation of DNMT1 stability through SET7-mediated lysine methylation in mammalian cells. Proc Natl Acad Sci U S A (2009) 106(13):5076–81. 10.1073/pnas.0810362106 19282482 PMC2654809

[71] Chim CS Pang R Fung TK Choi CL Liang R. Epigenetic dysregulation of Wnt signaling pathway in multiple myeloma. Leukemia (2007) 21(12):2527–36. 10.1038/sj.leu.2404939 17882284

[72] Dong C Wu Y Yao J Wang Y Yu Y Rychahou PG G9a interacts with snail and is critical for Snail-mediated E-cadherin repression in human breast cancer. J Clin Invest (2012) 122(4):1469–86. 10.1172/JCI57349 22406531 PMC3314447

[73] Feinberg AP Vogelstein B. Hypomethylation distinguishes genes of some human cancers from their normal counterparts. Nature (1983) 301(5895):89–92. 10.1038/301089a0 6185846

[74] Zhong X Chen X Guan X Zhang H Ma Y Zhang S Overexpression of G9a and MCM7 in oesophageal squamous cell carcinoma is associated with poor prognosis. Histopathology (2015) 66(2):192–200. 10.1111/his.12456 24805087

[75] Cerami E Gao J Dogrusoz U Gross BE Sumer SO Aksoy BA The cBio cancer genomics portal: an open platform for exploring multidimensional cancer genomics data. Cancer Discov (2012) 2(5):401–4. 10.1158/2159-8290.CD-12-0095 22588877 PMC3956037

[76] Gao J Aksoy BA Dogrusoz U Dresdner G Gross B Sumer SO Integrative analysis of complex cancer genomics and clinical profiles using the cBioPortal. Sci Signal (2013) 6(269):l1. 10.1126/scisignal.2004088 23550210 PMC4160307

[77] Lehnertz B Pabst C Su L Miller M Liu F Yi L The methyltransferase G9a regulates HoxA9-dependent transcription in AML. Genes Dev (2014) 28(4):317–27. 10.1101/gad.236794.113 24532712 PMC3937511

[78] Hua KT Wang MY Chen MW Wei LH Chen CK Ko CH The H3K9 methyltransferase G9a is a marker of aggressive ovarian cancer that promotes peritoneal metastasis. Mol Cancer (2014) 13:189. 10.1186/1476-4598-13-189 25115793 PMC4260797

[79] Wozniak RJ Klimecki WT Lau SS Feinstein Y Futscher BW. 5-Aza-2’-deoxycytidine-mediated reductions in G9A histone methyltransferase and histone H3 K9 di-methylation levels are linked to tumor suppressor gene reactivation. Oncogene (2007) 26(1):77–90. 10.1038/sj.onc.1209763 16799634

[80] Mechanic S Raynor K Hill JE Cowin P. Desmocollins form a distinct subset of the cadherin family of cell adhesion molecules. Proc Natl Acad Sci U S A (1991) 88(10):4476–80. 10.1073/pnas.88.10.4476 2034686 PMC51683

[81] Zou Z Anisowicz A Hendrix MJ Thor A Neveu M Sheng S Maspin, a serpin with tumor-suppressing activity in human mammary epithelial cells. Science (1994) 263(5146):526–9. 10.1126/science.8290962 8290962

[82] Lee SH Kim J Kim WH Lee YM. Hypoxic silencing of tumor suppressor RUNX3 by histone modification in gastric cancer cells. Oncogene (2009) 28(2):184–94. 10.1038/onc.2008.377 18850007

[83] Hasan NM Adams GE Joiner MC Marshall JF Hart IR. Hypoxia facilitates tumour cell detachment by reducing expression of surface adhesion molecules and adhesion to extracellular matrices without loss of cell viability. Br J Cancer (1998) 77(11):1799–805. 10.1038/bjc.1998.299 9667649 PMC2150343

[84] Ke XX Zhang D Zhu S Xia Q Xiang Z Cui H. Inhibition of H3K9 methyltransferase G9a repressed cell proliferation and induced autophagy in neuroblastoma cells. PLoS One (2014) 9(9):e106962. 10.1371/journal.pone.0106962 25198515 PMC4157855

[85] Li KC Hua KT Lin YS Su CY Ko JY Hsiao M Inhibition of G9a induces DUSP4-dependent autophagic cell death in head and neck squamous cell carcinoma. Mol Cancer (2014) 13:172. 10.1186/1476-4598-13-172 25027955 PMC4107555

[86] Yang Q Lu Z Singh D Raj JU. BIX-01294 treatment blocks cell proliferation, migration and contractility in ovine foetal pulmonary arterial smooth muscle cells. Cell Prolif (2012) 45(4):335–44. 10.1111/j.1365-2184.2012.00828.x 22691107 PMC3649875

[87] Ding J Li T Wang X Zhao E Choi JH Yang L The histone H3 methyltransferase G9A epigenetically activates the serine-glycine synthesis pathway to sustain cancer cell survival and proliferation. Cell Metab (2013) 18(6):896–907. 10.1016/j.cmet.2013.11.004 24315373 PMC3878056

[88] Laplante M Sabatini DM. mTOR signaling in growth control and disease. Cell (2012) 149(2):274–93. 10.1016/j.cell.2012.03.017 22500797 PMC3331679

[89] Kubicek S O’Sullivan RJ August EM Hickey ER Zhang Q Teodoro ML Reversal of H3K9me2 by a small-molecule inhibitor for the G9a histone methyltransferase. Mol Cell (2007) 25(3):473–81. 10.1016/j.molcel.2007.01.017 17289593

[90] Chang Y Zhang X Horton JR Upadhyay AK Spannhoff A Liu J Structural basis for G9a-like protein lysine methyltransferase inhibition by BIX-01294. Nat Struct Mol Biol (2009) 16(3):312–7. 10.1038/nsmb.1560 19219047 PMC2676930

[91] Savickiene J Treigyte G Stirblyte I Valiuliene G Navakauskiene R. Euchromatic histone methyltransferase 2 inhibitor, BIX-01294, sensitizes human promyelocytic leukemia HL-60 and NB4 cells to growth inhibition and differentiation. Leuk Res (2014) 38(7):822–9. 10.1016/j.leukres.2014.04.003 24832370

[92] Ueda J Ho JC Lee KL Kitajima S Yang H Sun W The hypoxia-inducible epigenetic regulators Jmjd1a and G9a provide a mechanistic link between angiogenesis and tumor growth. Mol Cell Biol (2014) 34(19):3702–20. 10.1128/MCB.00099-14 25071150 PMC4187738

[93] Vedadi M Barsyte-Lovejoy D Liu F Rival-Gervier S Allali-Hassani A Labrie V A chemical probe selectively inhibits G9a and GLP methyltransferase activity in cells. Nat Chem Biol (2011) 7(8):566–74. 10.1038/nchembio.599 21743462 PMC3184254

[94] Artal-Martinez de Narvajas A Gomez TS Zhang JS Mann AO Taoda Y Gorman JA Epigenetic regulation of autophagy by the methyltransferase G9a. Mol Cell Biol (2013) 33(20):3983–93. 10.1128/MCB.00813-13 23918802 PMC3811684

[95] Kim Y Kim YS Kim DE Lee JS Song JH Kim HG BIX-01294 induces autophagy-associated cell death via EHMT2/G9a dysfunction and intracellular reactive oxygen species production. Autophagy (2013) 9(12):2126–39. 10.4161/auto.26308 24322755

[96] Liu F Barsyte-Lovejoy D Li F Xiong Y Korboukh V Huang XP Discovery of an in vivo chemical probe of the lysine methyltransferases G9a and GLP. J Med Chem (2013) 56(21):8931–42. 10.1021/jm401480r 24102134 PMC3880643

[97] Jackson-Grusby L Beard C Possemato R Tudor M Fambrough D Csankovszki G Loss of genomic methylation causes p53-dependent apoptosis and epigenetic deregulation. Nat Genet (2001) 27(1):31–9. 10.1038/83730 11137995

[98] Yu Y Zeng P Xiong J Liu Z Berger SL Merlino G. Epigenetic drugs can stimulate metastasis through enhanced expression of the pro-metastatic Ezrin gene. PLoS One (2010) 5(9):e12710. 10.1371/journal.pone.0012710 20856924 PMC2938331

[99] Kaminskas E Farrell AT Wang YC Sridhara R Pazdur R. FDA drug approval summary: azacitidine (5-azacytidine, Vidaza) for injectable suspension. Oncologist (2005) 10(3):176–82. 10.1634/theoncologist.10-3-176 15793220

[100] Garcia-Manero G Yang H Bueso-Ramos C Ferrajoli A Cortes J Wierda WG Phase 1 study of the histone deacetylase inhibitor vorinostat (suberoylanilide hydroxamic acid [SAHA]) in patients with advanced leukemias and myelodysplastic syndromes. Blood (2008) 111(3):1060–6. 10.1182/blood-2007-06-098061 17962510

[101] Modesitt SC Sill M Hoffman JS Bender DP Gynecologic Oncology G. A phase II study of vorinostat in the treatment of persistent or recurrent epithelial ovarian or primary peritoneal carcinoma: a gynecologic oncology group study. Gynecol Oncol (2008) 109(2):182–6. 10.1016/j.ygyno.2008.01.009 18295319

[102] Ramalingam SS Parise RA Ramanathan RK Lagattuta TF Musguire LA Stoller RG Phase I and pharmacokinetic study of vorinostat, a histone deacetylase inhibitor, in combination with carboplatin and paclitaxel for advanced solid malignancies. Clin Cancer Res (2007) 13(12):3605–10. 10.1158/1078-0432.CCR-07-0162 17510206

[103] Falahi F van Kruchten M Martinet N Hospers GA Rots MG. Current and upcoming approaches to exploit the reversibility of epigenetic mutations in breast cancer. Breast Cancer Res (2014) 16(4):412. 10.1186/s13058-014-0412-z 25410383 PMC4303227

[104] Gibbons GS Owens SR Fearon ER Nikolovska-Coleska Z. Regulation of Wnt signaling target gene expression by the histone methyltransferase DOT1L. ACS Chem Biol (2015) 10(1):109–14. 10.1021/cb500668u 25361163

[105] Liu TP Lo HL Wei LS Hsiao HH Yang PM. *S*-Adenosyl-L-methionine-competitive inhibitors of the histone methyltransferase EZH2 induce autophagy and enhance drug sensitivity in cancer cells. Anticancer Drugs (2015) 26(2):139–47. 10.1097/CAD.0000000000000166 25203626 PMC4276571

[106] Kudithipudi S Kusevic D Jeltsch A. Non-radioactive protein lysine methyltransferase microplate assay based on reading domains. ChemMedChem (2014) 9(3):554–9. 10.1002/cmdc.201300111 23671032

[107] Rose NR Woon EC Tumber A Walport LJ Chowdhury R Li XS Plant growth regulator daminozide is a selective inhibitor of human KDM2/7 histone demethylases. J Med Chem (2012) 55(14):6639–43. 10.1021/jm300677j 22724510 PMC4673902

[108] Suzuki T Ozasa H Itoh Y Zhan P Sawada H Mino K Identification of the KDM2/7 histone lysine demethylase subfamily inhibitor and its antiproliferative activity. J Med Chem (2013) 56(18):7222–31. 10.1021/jm400624b 23964788 PMC3929130

[109] Hamada S Kim TD Suzuki T Itoh Y Tsumoto H Nakagawa H Synthesis and activity of N-oxalylglycine and its derivatives as Jumonji C-domain-containing histone lysine demethylase inhibitors. Bioorg Med Chem Lett (2009) 19(10):2852–5. 10.1016/j.bmcl.2009.03.098 19359167

[110] Heinemann B Nielsen JM Hudlebusch HR Lees MJ Larsen DV Boesen T Inhibition of demethylases by GSK-J1/J4. Nature (2014) 514(7520):E1–2. 10.1038/nature13688 25279926

[111] Schiller R Scozzafava G Tumber A Wickens JR Bush JT Rai G A cell-permeable ester derivative of the JmjC histone demethylase inhibitor IOX1. ChemMedChem (2014) 9(3):566–71. 10.1002/cmdc.201300428 24504543 PMC4503230

[112] Rai G Kawamura A Tumber A Liang Y Vogel JL Arbuckle JH Discovery of ML324, a JMJD2 demethylase inhibitor with demonstrated antiviral activity. Probe Reports from the NIH Molecular Libraries Program. Bethesda, MD (2010). 24260783

[113] Yang M Culhane JC Szewczuk LM Jalili P Ball HL Machius M Structural basis for the inhibition of the LSD1 histone demethylase by the antidepressant trans-2-phenylcyclopropylamine. Biochemistry (2007) 46(27):8058–65. 10.1021/bi700664y 17569509

[114] Schenk T Chen WC Gollner S Howell L Jin L Hebestreit K Inhibition of the LSD1 (KDM1A) demethylase reactivates the all-trans-retinoic acid differentiation pathway in acute myeloid leukemia. Nat Med (2012) 18(4):605–11. 10.1038/nm.2661 22406747 PMC3539284

[115] Konovalov S Garcia-Bassets I. Analysis of the levels of lysine-specific demethylase 1 (LSD1) mRNA in human ovarian tumors and the effects of chemical LSD1 inhibitors in ovarian cancer cell lines. J Ovarian Res (2013) 6(1):75. 10.1186/1757-2215-6-75 24165091 PMC4176291

[116] Wang Z Yang D Zhang X Li T Li J Tang Y Hypoxia-induced down-regulation of neprilysin by histone modification in mouse primary cortical and hippocampal neurons. PLoS One (2011) 6(4):e19229. 10.1371/journal.pone.0019229 21559427 PMC3084787

[117] Wang L Chang J Varghese D Dellinger M Kumar S Best AM A small molecule modulates Jumonji histone demethylase activity and selectively inhibits cancer growth. Nat Commun (2013) 4:2035. 10.1038/ncomms3035 23792809 PMC3724450

[118] Holmlund T Lindberg MJ Grander D Wallberg AE. GCN5 acetylates and regulates the stability of the oncoprotein E2A-PBX1 in acute lymphoblastic leukemia. Leukemia (2013) 27(3):578–85. 10.1038/leu.2012.265 23044487

[119] Milite C Feoli A Sasaki K La Pietra V Balzano AL Marinelli L A novel cell-permeable, selective, and noncompetitive inhibitor of KAT3 histone acetyltransferases from a combined molecular pruning/classical isosterism approach. J Med Chem (2015) 58(6):2779–98. 10.1021/jm5019687 25730130

[120] Lau OD Kundu TK Soccio RE Ait-Si-Ali S Khalil EM Vassilev A HATs off: selective synthetic inhibitors of the histone acetyltransferases p300 and PCAF. Mol Cell (2000) 5(3):589–95. 10.1016/S1097-2765(00)80452-9 10882143

[121] Balasubramanyam K Varier RA Altaf M Swaminathan V Siddappa NB Ranga U Curcumin, a novel p300/CREB-binding protein-specific inhibitor of acetyltransferase, represses the acetylation of histone/nonhistone proteins and histone acetyltransferase-dependent chromatin transcription. J Biol Chem (2004) 279(49):51163–71. 10.1074/jbc.M409024200 15383533

[122] Mantelingu K Reddy BA Swaminathan V Kishore AH Siddappa NB Kumar GV Specific inhibition of p300-HAT alters global gene expression and represses HIV replication. Chem Biol (2007) 14(6):645–57. 10.1016/j.chembiol.2007.04.011 17584612

[123] Oike T Komachi M Ogiwara H Amornwichet N Saitoh Y Torikai K C646, a selective small molecule inhibitor of histone acetyltransferase p300, radiosensitizes lung cancer cells by enhancing mitotic catastrophe. Radiother Oncol (2014) 111(2):222–7. 10.1016/j.radonc.2014.03.015 24746574

[124] Xu LX Li ZH Tao YF Li RH Fang F Zhao H Histone acetyltransferase inhibitor II induces apoptosis in glioma cell lines via the p53 signaling pathway. J Exp Clin Cancer Res (2014) 33(1):108. 10.1186/s13046-014-0108-3 25523932 PMC4321714

[125] Ye X Yuan L Zhang L Zhao J Zhang CM Deng HY. Garcinol, an acetyltransferase inhibitor, suppresses proliferation of breast cancer cell line MCF-7 promoted by 17beta-estradiol. Asian Pac J Cancer Prev (2014) 15(12):5001–7. 10.7314/APJCP.2014.15.12.5001 24998578

[126] Butler LM Webb Y Agus DB Higgins B Tolentino TR Kutko MC Inhibition of transformed cell growth and induction of cellular differentiation by pyroxamide, an inhibitor of histone deacetylase. Clin Cancer Res (2001) 7(4):962–70. 11309347

[127] Denis I El Bahhaj F Collette F Delatouche R Gueugnon F Pouliquen D Histone deacetylase inhibitor-polymer conjugate nanoparticles for acid-responsive drug delivery. Eur J Med Chem (2015) 95:369–76. 10.1016/j.ejmech.2015.03.037 25827403

[128] Meidhof S Brabletz S Lehmann W Preca BT Mock K Ruh M ZEB1-associated drug resistance in cancer cells is reversed by the class I HDAC inhibitor mocetinostat. EMBO Mol Med (2015) 7(6):831–47. 10.15252/emmm.201404396 25872941 PMC4459821

[129] Richon VM Webb Y Merger R Sheppard T Jursic B Ngo L Second generation hybrid polar compounds are potent inducers of transformed cell differentiation. Proc Natl Acad Sci U S A (1996) 93(12):5705–8. 10.1073/pnas.93.12.5705 8650156 PMC39124

[130] Campbell GR Bruckman RS Chu YL Spector SA. Autophagy induction by histone deacetylase inhibitors inhibits HIV type 1. J Biol Chem (2015) 290(8):5028–40. 10.1074/jbc.M114.605428 25540204 PMC4335239

[131] Rosato RR Almenara JA Maggio SC Coe S Atadja P Dent P Role of histone deacetylase inhibitor-induced reactive oxygen species and DNA damage in LAQ-824/fludarabine antileukemic interactions. Mol Cancer Ther (2008) 7(10):3285–97. 10.1158/1535-7163.MCT-08-0385 18852132 PMC2586957

[132] Choy E Flamand Y Balasubramanian S Butrynski JE Harmon DC George S Phase 1 study of oral abexinostat, a histone deacetylase inhibitor, in combination with doxorubicin in patients with metastatic sarcoma. Cancer (2015) 121(8):1223–30. 10.1002/cncr.29175 25536954 PMC4393337

[133] Ryu H Smith K Camelo SI Carreras I Lee J Iglesias AH Sodium phenylbutyrate prolongs survival and regulates expression of anti-apoptotic genes in transgenic amyotrophic lateral sclerosis mice. J Neurochem (2005) 93(5):1087–98. 10.1111/j.1471-4159.2005.03077.x 15934930

[134] Kang SH Seok YM Song MJ Lee HA Kurz T Kim I. Histone deacetylase inhibition attenuates cardiac hypertrophy and fibrosis through acetylation of mineralocorticoid receptor in spontaneously hypertensive rats. Mol Pharmacol (2015) 87(5):782–91. 10.1124/mol.114.096974 25667225

[135] Frys S Simons Z Hu Q Barth MJ Gu JJ Mavis C Entinostat, a novel histone deacetylase inhibitor is active in B-cell lymphoma and enhances the anti-tumor activity of rituximab and chemotherapy agents. Br J Haematol (2015) 169(4):506–19. 10.1111/bjh.13318 25712263 PMC5802357

[136] Rabizadeh E Merkin V Belyaeva I Shaklai M Zimra Y. Pivanex, a histone deacetylase inhibitor, induces changes in BCR-ABL expression and when combined with STI571, acts synergistically in a chronic myelocytic leukemia cell line. Leuk Res (2007) 31(8):1115–23. 10.1016/j.leukres.2006.12.015 17267032

